# Novel Imidazole Aldoximes with Broad-Spectrum Antimicrobial Potency against Multidrug Resistant Gram-Negative Bacteria

**DOI:** 10.3390/molecules23051212

**Published:** 2018-05-18

**Authors:** Mirjana Skočibušić, Renata Odžak, Alma Ramić, Tomislav Smolić, Tomica Hrenar, Ines Primožič

**Affiliations:** 1 Department of Biology, Faculty of Science, University of Split, R. Boškovića 33, HR-21 000 Split, Croatia; 2 Department of Chemistry, Faculty of Science, University of Split, R. Boškovića 33, HR-21 000 Split, Croatia; rodzak@pmfst.hr; 3 Department of Chemistry, Faculty of Science, University of Zagreb, Horvatovac 102a, HR-10 000 Zagreb, Croatia; alma.ramic@gmail.com (A.R.); Tom_Smolic@cargill.com (T.S.); tomica.hrenar@chem.pmf.hr (T.H.)

**Keywords:** imidazole 2-aldoximes, quaternary imidazolium salts, antimicrobial activity, extended-spectrum β-lactamase (ESBL), multivariate analysis, multidrug resistance

## Abstract

In the search for a new class of potential antimicrobial agents, five novel *N*-substituted imidazole 2-aldoximes and their six quaternary salts were evaluated. The antimicrobial activity was assessed against a panel of representative Gram-positive and Gram-negative bacteria, including multidrug resistant bacteria. All compounds demonstrated potent in vitro activity against the tested microorganisms, with MIC values ranging from 6.25 to 50.0 μg/mL. Among the tested compounds, two quaternary compounds (*N*-but-3-enyl- and *meta*- (**10**) or *para*- *N*-chlorobenzyl (**11**) imidazolium 2-aldoximes) displayed the most potent and broad-spectrum activity against both Gram-positive and Gram-negative bacterial strains. The broth microdilution assay was also used to investigate the antiresistance efficacy of the both most active compounds against a set of *Enterobacteriaceae* isolates carried a multiple extended-spectrum β-lactamases (ESBLs) in comparison to eight clinically relevant antibiotics. *N*-but-3-enyl-*N-meta*-chlorobenzyl imidazolium 2-aldoxime was found to possess promising antiresistance efficacy against a wide range of β-lactamases producing strains (MIC 2.0 to 16.0 μg/mL). Best results for that compound were obtained against *Escherichia coli* and *Enterobacter cloacae* producing multiple β-lactamases form A and C molecular classes, which were 32- and 128-fold more potent than ceftazidime and cefotaxime, respectively. To visualize the results, principal component analysis was used as an additional classification tool. The mixture of ceftazidime and compound **10** (3 μg:2 μg) showed a strong activity and lower the necessary amount (up to 40-fold) of **10** against five of ESBL-producing isolates (MIC ≤ 1 µg/mL).

## 1. Introduction

Over the past decade, the widespread emergence of antibiotic drug resistance has resulted in a worldwide health crisis of global dimensions. The continuous emergence and proliferation of pathogenic bacteria resistant to many or all current antibiotics is a major public health concern and one of the particular importance in clinical settings. The most recent World Economic Forum Global Risks reports [1] have listed antibiotic resistance as one of the greatest threats to human health. Meanwhile, resistance rates around the world are rising, new resistance mechanisms are emerging, and infections caused by multidrug-resistant Gram-negative bacteria are becoming particularly difficult to treat.

The β-lactam antibiotics are essential for the treatment of a wide range of human bacterial diseases. Among clinically important Gram-negative bacteria, the production of β-lactamases is the most frequent factor contributing to β-lactam resistance [2]. The molecular classification of β-lactamase is based on the amino acid sequence and divides β-lactamases into class A, C and D enzymes which employ a serine residue as the nucleophilic species to attack the lactam carbonyl group, forming an acyl enzyme intermediate before hydrolysis [3]. Almost invariably, however, mutations or new enzymes have emerged that enable the recognition and hydrolysis of the latest generation of β-lactam antibiotics. A new group of class A extended-spectrum β-lactamases (ESBLs), called CTX-M enzymes, has emerged worldwide and it is now the most frequently observed extended-spectrum β-lactamases in several areas. CTX-M enzymes hydrolyse not only the penicillin, but also the first-, second and third-generation cephalosporins and the current lack of effective treatment options is the reason for increasing number of invasive bacterial infections caused by ESBL-producing pathogens [4]. Of great concern is the increasing ability of the bacteria to frequently harbour multiple β-lactamases with different substrate profiles, making virtually all β-lactam antibiotics ineffective.

To restore their antibacterial activity against Gram-negative pathogens, β-lactams have been paired with inhibitors of β-lactamases. The currently available, of the three classical β-lactamase inhibitors such as clavulanic acid, sulbactam and tazobactam, are derived from β-lactam scaffolds, are now met with an increasingly prevalent panel of inhibitor-resistant bacterial strains [5]. Unfortunately, β-lactamases have also evolved resistance to inhibitors; point mutations in *bla*_TEM_ and *bla*_SHV_ gave rise to amino acid substitutions in the parent enzyme that result in inhibitor resistant β-lactamases causing elevated minimum inhibitory concentration (MIC) to β-lactam/β-lactamase inhibitor combinations. These limitations, as well as the widespread bacterial production of multiple β-lactamases including class-A, class-C ESBLs has motivated several research groups to search for new inhibitors with a non-lactam structural scaffold as well with a broad-spectrum inhibition profile [6,7].

**Figure 1:**
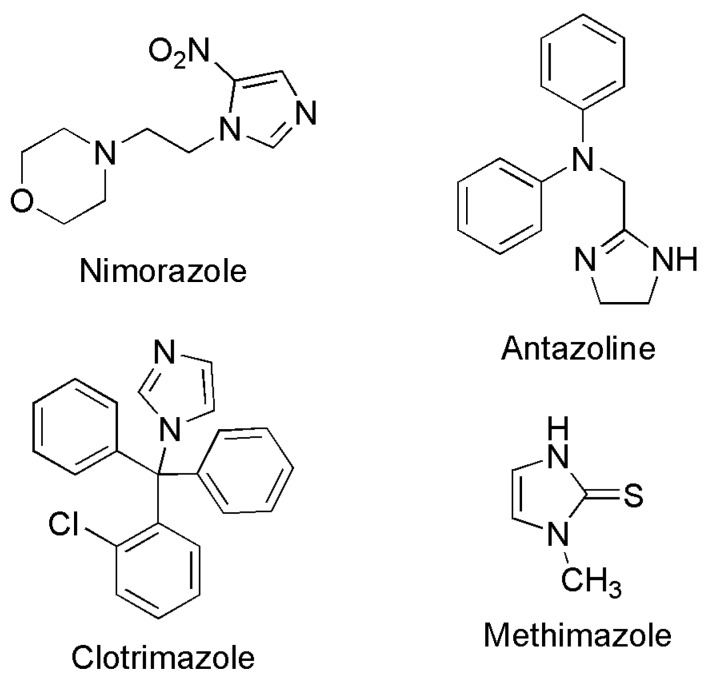
Some pharmaceuticals having imidazole cores.

Imidazole is a five-membered aromatic heterocycle which contains two nitrogen atoms and is present in the structure of many different molecules and intermediates of biological significance, including purines, many cofactors, and histidine and synthetic bioactive molecules [8]. The imidazole ring is amphoteric and highly polar, can easily accept or donate protons, readily form different weak interactions and has multiple binding sites which allows it to interact easily with the biopolymers of living systems. Based on several literature surveys, imidazole-based small molecules have been described as promising pharmaceutical agents in view of their unique bioactivity patterns, ranging from antioxidant, anti-inflammatory, analgesic, and neuroprotective to chemotherapeutic and anti-HIV activities [8,9,10,11]. Imidazoles and in particular their salts comprise a boundless and emerging field, due to not only their favourable properties such as excellent bioavailability, good tissue penetrability and permeability but also a relatively low incidence of adverse and toxic effects [12]. So far, many imidazole-based derivatives (Figure 1) have been successfully developed and employed as clinical drugs [13].

Metronidazole, a nitroimidazole compound, is one of the most commonly used antimicrobial agents, which was initially found to be effective particularly for the treatment of parasitic infections. Since then, this compound has also played an important role for the treatment of infections caused by a variety of anaerobic and microaerophilic bacteria, including *Helicobacter pylori* which has been strongly associated with gastritis and duodenal ulcers [14]. In addition, it has also been recognized [15] that many imidazole-containing ligands could exhibit large chemical-shift variations when bound to a molecular target, such as a protein, offering valuable information about changes in the local structure of the ligand or target. Among others, imidazole core structures are found in different carboxypeptidase, hemeoxygenase and β-lactamase inhibitors [16,17] as well as among compounds with anti-inflammatory, anticancer, antibacterial, antifungal, antitubercular, antidiabetic and antiviral activity [18,19,20,21,22,23].

Additionally, oximes and their complexes caught our attention, as they appear to be due not only amenable to biotransformation and conjugations with organic and inorganic molecules but also serve as the important templates of the novel antimicrobial drugs for the treatment of infections caused by wide range of multidrug-resistant Gram-negative pathogens [24]. Oximes have been recognized as privileged structures for many important pharmaceuticals and synthetic chemistry applications, and often act as chemical building blocks for the synthesis of some relevant antimicrobial and antihypertensive agents as well as insecticides [25,26]. Recently, the oxime scaffold has emerged as a pharmacophore of choice for designing antimicrobial agents active and has been successfully used for functionalized carbapenems are one of the most potent of antibacterial agents as well as are used as last resort against infections in the clinical field. Therefore, the structural modification of imidazole by introducing a 2-aldoximes fragment should be a highly attractive topic in the antibacterial drug discovery.

**Figure 2:**
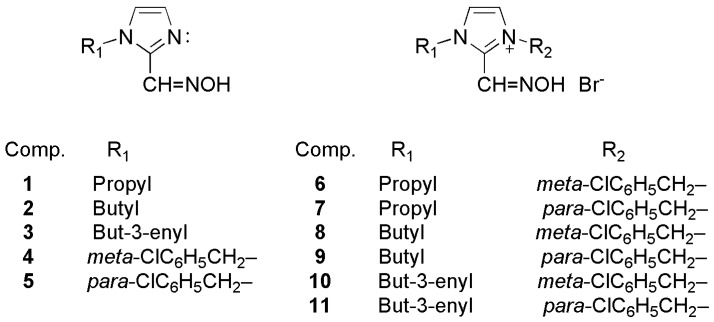
Structures of the tested imidazole 2-aldoximes.

We have recently described the potent in vitro antimicrobial efficacy of a series of substituted *N*-phenyl imidazole oximes and their quaternary salts against a panel of both clinically relevant antibiotic susceptible Gram-positive and antibiotic resistant Gram-negative bacteria that carry diverse resistance genes for the most commonly important antibiotics [24]. These results have encouraged further structural optimization studies to search for more potent antimicrobial agents particularly against Gram-negative pathogens producing a wide range of the serine β-lactamases based on the imidazole oxime scaffold. In this study, we have prepared series of novel *N*-substituted imidazoles having alkyl (propyl, butyl, but-3-enyl) or chlorobenzyl (*meta*-chlorobenzyl, *para*-chlorobenzyl) substituents at position 1 of an imidazole ring bearing a 2-aldoxime group to yield effective new antimicrobial agents. In addition, *N*-substituted imidazole 2-aldoximes were converted into their corresponding quaternary salts, Figure 2. In a preliminary screening, antimicrobial efficacy of all the synthesized imidazole 2-aldoximes was analysed by disc diffusion assay against the collections of both antibiotic susceptible Gram-positive and resistant Gram-negative bacteria in order to obtain their antimicrobial profiles. Additionally, their antimicrobial efficacy against Gram-negative multiple β-lactamases producing isolates are reported in the present work.

## 2. Results and Discussion

### 2.1. Chemistry

**Scheme 1:**
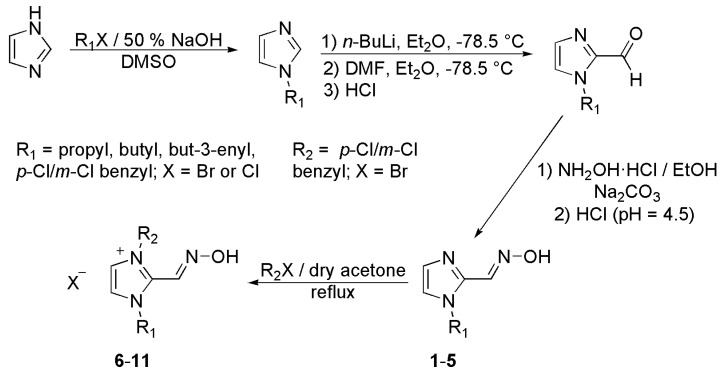
General procedure for the synthesis of imidazole derivatives.

*N*-substituted imidazole oximes were prepared according to reported procedures (Scheme 1) [27,28,29]. Briefly, commercially available imidazole was alkylated with appropriate alkyl or benzyl halide derivatives. Desired 2-carbaldehydes were prepared by the reaction with *n*-butyl lithium and dimethylformamide. Imidazole-2-carbaldehyde derivatives were transformed into oximes **1**–**5** with hydroxylamine in ethanol. Reaction of oximes **1**–**3** with the appropriate substituted benzyl bromides gave desired quaternary salts **6**–**11**.

### 2.2. Biological Evaluation

#### 2.2.1. Antimicrobial Activity

Antimicrobial efficacy of the synthesized imidazole 2-aldoximes, **1**–**5** and their corresponding quaternary compounds, **6**–**11** were evaluated by the disc diffusion assay against the collections of both antibiotic susceptible Gram-positive and Gram-negative bacteria in order to obtain their antimicrobial profiles. Activities of the target compounds were expressed as the mean diameter of the growth inhibition zone (mm) against these microorganisms and are listed in Table 1, along with the activity of the reference compound gentamicin (**GEN**).

Despite the structural differences, most of the imidazole 2-aldoximes derivatives demonstrated potent and broad-spectrum activity against selected clinically important pathogens with varying degrees of inhibition under that condition. The mean inhibition diameters of compounds were found in the range from 10.7 ± 0.6 to 21.8 ± 0.9 mm.

It is noteworthy that the tested compounds are not only active against Gram-positive bacteria, but also demonstrated significant antibacterial effects against Gram-negative bacteria as well. Among the Gram-negative bacteria tested, two strains, namely *Escherichia coli* (from 12.4 ± 0.71 to 21.8 ± 0.9 mm) and *Pseudomonas aeruginosa* (from 12.2 ± 1.1 to 20.2 ± 0.4 mm) showed relatively high sensitivity toward all tested compounds with considerable zones of growth inhibition. Among them, compound **6**, which has an *n*-propyl substituent at the nitrogen ring atom has been found as one of the most efficient imidazole 2-aldoxime derivative against *Escherichia coli* (20.7 ± 0.5 mm), *Klebsiella pneumoniae* (20.1 ± 0.8 mm) and *Pseudomonas aeruginosa* (20.2 ± 0.4 mm) strains. It is particularly important that the imidazole 2-aldoximes derivatives demonstrated potent activity against *P. aeruginosa* which is one of the most prevalent multidrug-resistant pathogen worldwide, exhibiting increasing resistance to the latest antibiotic therapies and responsible for 10% of hospital acquired infections resulting in significant morbidity and mortality [30].

In order to confirm the found antimicrobial efficacy, imidazole 2-aldoxime derivatives were then tested for activity against a panel of clinically relevant pathogens to determine MIC values by a broth microdilution method.

The antimicrobial bioassay results of the target compounds and the reference compound gentamicin are summarized in Table 2. It can be seen that all target imidazole 2-aldoxime derivatives demonstrated potent and broad spectrum activities against selected microorganisms with MIC values in the range from 6.25 to 50.0 μg/mL. In general, *N*-substituted imidazoles **1**–**5** showed similar potent antimicrobial activities against a representative panel of the Gram-positive and Gram-negative bacteria tested (MIC = 12.5–50.0 μg/mL). Compounds **1** and **4** were found to possess not only excellent activity against Gram-positive *Enterococcus faecalis* and *Staphylococcus aureus*, but also strong inhibitory activity against Gram-negative *Escherichia coli* with equal MIC (12.50 μg/mL) which is to three-fold more active than the standard antibiotic gentamicin. Compounds **1**, **3** and **4** also showed promising inhibitory profiles against clinically important *Pseudomonas aeruginosa* (25 µg/mL) which is approximately three-fold lower than the positive control gentamicin.

Furthermore, the data showed that, in general, *N*-substituted imidazole 2-aldoximes showed very similar activity in inhibiting bacterial growth as quaternary imidazolium salts for Gram-positive and Gram-negative bacteria with exception of compounds **10** and **11**. Although possessing similar structural elements as those in compounds **3**, **4** and **5,** the presence of both electron rich groups are required for better potency and spectrum of antimicrobial activities.

However, compared with other compounds minimum inhibitory concentrations demonstrated that *m*-chlorobenzyl derivative **10** was found to be the most superior compound, especially for Gram-negative bacteria (*Escherichia coli*, *Klebsiella pneumoniae* and *Pseudomonas aeruginosa*) and exhibited of MIC values in the range from 12.5 even to 6.25 μg/mL. This compound also showed promising antimicrobial activities especially against *Escherichia coli* and *Klebsiella pneumoniae* with equal MIC values of 6.25 μg/mL, which was superior to gentamicin.

Most promising compounds **10** and **11** were therefore selected for further MIC experiments against a panel of Gram-negative isolates producing a wide range of the extended-spectrum β-lactamases (ESBLs).

#### 2.2.2. Activity toward Gram-Negative β-Lactamase Producing Strains

In the present study, we also performed an extensive in vitro screening of both quaternary but-3-enyl compounds **10** and **11** against genetically diverse Gram-negative β-lactamase producing strains carried a wide variety of ESBLs to overcome β-lactamase mediated resistance. For two compounds, **10** and **11** antimicrobial profiles have been determined since they demonstrated the strongest efficacy against a wide range of Gram-negative β-lactamase-producing isolates with a MIC values in the range from 6.25 to 25.00 μg/mL and 12.50 to 25.00 μg/mL, respectively (Table 2).

Antimicrobial activity of both tested compounds was investigated by the determination of the minimum inhibitory concentrations (MICs) in comparison with the different classes of antibiotics with diverse mechanisms of action (Table 3). The MICs of relevant antibacterial agents including ceftazidime, cefotaxime, ciprofloxacin, gentamicin, tetracycline, tobramycin, imipenem and meropenem are shown to illustrate the types of resistance associated with the each phenotype and genotype profiles and indicates the levels and profiles of resistance in the isolates tested. The microdilution assays were performed against ten *Enterobacteriaceae* strains (2 *Klebsiella pneumoniae*, 3 *Escherichia coli*, 1 *Citrobacter freundii*, 2 *Enterobacter cloacae*, 1 *Raultella terrigena* and 1 *Pantoea agglomerans*), all with known resistance mechanisms such as various combinations of extended-spectrum β-lactamases of both class A and C phenotypically and molecularly determined.

As shown in Table 3, both tested compounds **10** and **11** showed significant antibacterial potency against a wide variety of ESBL-producing strains when compared to eight broad spectrum clinically relevant antibiotics, with MICs values ranging from 2 to 32 μg/mL. Compound **10** was found to possess promising antimicrobial efficacy against Escherichia coli and Enterobacter cloacae producing multiple β-lactamases from A and C molecular classes which was 32-fold and 128-fold more potent than ceftazidime and cefotaxime, respectively. Compound **10** also demonstrated potent activity by inhibiting the multiple β-lactamases in isolates of Klebsiella pneumoniae, Escherichia coli and Raultella terrigena (MIC = 4 μg/mL), produced clinically relevant class A and C β-lactamases such as TEM-1, CTX-15 and AmpC enzymes that are poorly inhibited by clavulanic acid with a MIC value of 8 μg/mL. Moreover, the promising inhibitory activity toward a wide range of β-lactamase-producing Gram-negative strains of compound **10** was also seen against TEM-1, CTX-M-15 producing K. pneumoniae, and against TEM-1, CTX-M-15, AmpC-overproducing Citrobacter freundii, with a MIC of 8 μg/mL. It is however interesting to note that compound **10** displayed potent and board spectrum activity against Escherichia coli producing TEM-1, SHV-12 and Enterobacter cloacae producing TEM-1, SHV-12, CTX-15 β-lactamases (MIC = 2 μg/mL) than ceftazidime, cefotaxime, gentamicin, tetracycline and tobramycin (Table 3). Those results demonstrated the antimicrobial efficacy of both **10** and **11** compounds differed depending on the isolates and their β-lactamase expression profiles and that the compounds operate by a different mode of action and have not been affected by the resistance mutations present in tested strains.

### 2.3. Principal Component Analysis

**Figure 3:**
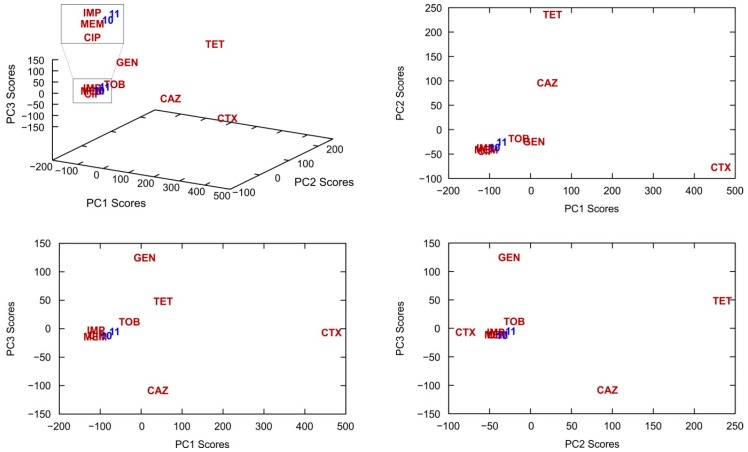
Classification of compounds **10** and **11** and tested standard antibiotics calculated by PCA performed on the mean-centered covariance matrix of their MIC values.

To classify compounds **10** and **11** and tested standard antibiotics according to their activities (Table 3), we performed principal component analysis (PCA) on the mean-centered covariance matrix of MIC values. PCA was used exclusively for classification purpose and visualization of the investigated set of data presented in Table 3. The first three principal components explained more than 96% of the total variance among the investigated data. Therefore, we used 3D classification model presented in Figure 3 (together with all 2D projections). It was shown that compounds **10** and **11** have the most similar antimicrobial activity in comparison to imiperem and meropenem, which are the broad-spectrum β-lactam antibiotics (subgroup of carbapenems).

### 2.4. Combination Study of Compound 10 with Ceftazidime

In order to evaluate effects of the most active compound **10** to β-lactam antibiotic ceftazidime against a variety of ESBL-producing isolates, the various combinations of compound **CAZ** and **10** were investigated. Among tested combinations, the ratio 3:2 (**CAZ**:**10**) was found as the one with the best activity which is greater than the sum of the effects produced by the components alone. As shown in Table 4, compound **10** significantly improved the activity of ceftazidime (for *Escherichia coli*, SHV-12, CTX-15 more then 200-fold) against a variety of ESBL-producing isolates, restoring susceptibility of even high level resistance isolates with multiple β-lactamases. The present data also demonstrated the strong effect of ceftazidime on compound **10**, lowering the amount of **10** essential to obtain strong antibiotic response. The addition of ceftazidime to the compound **10** resulted in a decrease of necessary amount of **10** from 5- to 40-fold, the most reduced amount observed for *Pantoea agglomerans*, CTX-15.

## 3. Materials and Methods

### 3.1. General Information

All the synthesized compounds [29] were evaluated for their in vitro antibacterial activity. The tested microorganisms were obtained from the culture collection at the American Type Culture Collection (ATCC, Rockville, MD, USA) and at the Microbiology laboratory, Department of Biology, Faculty of Natural Science, University of Split, Croatia (FNSST). The assayed collection included four Gram-positive bacteria *Bacillus cereus* (ATTC 11778), *Enterococcus faecalis* (ATCC 29212), and *Staphylococcus aureus* (ATCC 25923) *Clostridium perfringens* (FNSST 4999) and four Gram-negative ampicillin-resistant bacterial strains *Escherichia coli* (FNSST 982), *Klebsiella pneumoniae* (FNSST 011), *Pseudomonas aeruginosa* (FNSST 982) and *Chronobacter sakazakii* (FNSST 014). Bacterial strains were cultured overnight at 37 °C in tryptic soy broth (TSB) to achieve optical densities corresponding to 10^6^ colony forming units (cfu/mL) for bacterial strains.

### 3.2. Disc Diffusion Assay

In order to investigate the antimicrobial activities of the synthesized compounds, a disc diffusion assay was employed according to the CLSI guidelines. Briefly, 100 µL of suspension containing 10^6^ colony-forming units (cfu/mL) of bacterial cells was spread on a Mueller Hinton agar (Becton Dickinson, Sparks, MD, USA). The stock solutions of synthesized compounds were prepared by dissolving in DMSO to a final concentration of 10 mg/mL. The sterile filter discs (6 mm) were individually loaded with 20 µL of the stock solution, equivalent to a final concentration at 200 µg/disc of synthesized compounds and then placed on the nutrient agar that had been previously inoculated with the target microbial strains. Additionally, DMSO was used as a negative control, and gentamicin (15 μg) was used as positive controls. The plates were incubated for overnight at 37 °C for bacterial strains. Antibacterial activity was assessed by measuring the diameter of the inhibition zone in millimetres, including disc diameter for the test isolates, compared to the controls. Samples were assayed in triplicate for each condition and the diameter of inhibition zones were presented as mean ± SE values.

### 3.3. Minimum Inhibitory Concentration Assay

In addition, antimicrobial activities of the synthesized compounds were also tested by a broth microdilution assay in 96 well plates. The standard two-fold serial microdilution assay described by the Clinical and Laboratory Standards Institute was performed for the assessment of the minimum inhibitory concentrations (MICs). Bacteria were grown overnight in Mueller-Hilton broth (MHB) at 37 °C. The microbial cultures were diluted in fresh MHB to a final concentration of 10^6^ CFU/mL for bacteria. Initially the target compounds were dissolved in DMSO to prepare the stock solutions of 10 mg/mL, then the tested compounds and reference drugs were diluted to obtain concentrations ranging from 200, 100, 50, 25, 12.5, 6.25, 3.12, 1.56 to 0.78 μg/mL in Mueller-Hinton broth (MHB).

Serial dilutions of the synthesized compounds and reference drug were added to the microtiter plates in a volume of 100 µL. Each well was additionally inoculated with 10 µL of the target microorganism and incubated at 37 °C for 18–24 h. The MIC value was determined as the lowest concentration of the sample at which the tested microorganisms did not demonstrate any visible growth after incubation. As an indicator of bacterial growth, 50 μL of 0.2 mg/mL *p*-iodonitrotetrazoliumchloride (INT; Sigma-Aldrich Co. Ltd., Poole, UK) was added to the wells and incubated at 37 °C for 30 min. Following addition of INT and incubation, the MIC was determined as the lowest sample concentration at which no pink colour appeared. In this study, no bioactivity was defined as a MIC > 1000 µg/mL, mild bioactivity as a MIC in the range 512–1000 µg/mL, moderate bioactivity as a MIC in the range 128–512 µg/mL, good bioactivity as a MIC in the range 32–128 µg/mL, strong bioactivity as a MIC in the range 10–32 µg/mL and very strong bioactivity as a MIC < 10 µg/mL.

### 3.4. Principal Component Analysis

Statistical analysis were performed using the second order tensor analysis tool principal component analysis (PCA) where data matrix (or two-way array) ***X*** of rank *r* with mean centered columns that consists of *i* rows (compunds) and *j* variables (MIC values) is decomposed as a sum of *r* matrices $t_{i}p_{i}^{\tau }$ with rank 1

$$X=\overset{r}{\underset{i=1}{\sum }}t_{i}p_{i}^{\tau }$$

where $t_{i}$ stand for score and $p_{i}^{\tau }$ for loading vectors. PCA finds the best linear projections for a high dimensional set of data in the least squares sense. Scores represent projections of the original points on the principal component direction and can be used for classification, whereas loadings represent eigenvectors of data covariance (or correlation) matrix and can be used for the identification of variability among the data. Ideas of PCA go back to Beltrami [31] and Pearson [32] while the name was introduced by Hotelling [33]. More details can be found in the recent literature [34,35].

Minimum inhibitory concentration data were exported to the ASCII format and arranged in the matrix ***X*** (numbers written in a free format). Data were mean-centered and PCA on the covariance matrix was carried out using our own multivariate analysis code [36] based on the NIPALS algorithm [37].

## 4. Conclusions

Although the imidazole aldoxime scaffold is not common in the antimicrobial agent literature, our previous study [24] and the novel library of imidazole 2-aldoxime derivatives presented in this paper show that there is a huge potential for the design of a compounds effective against a wide range of clinically relevant Gram-negative pathogens carrying multiple β-lactamases from the A and C molecular classes. All compounds showed good to strong broad-spectrum activity against a representative panel of the Gram-positive and Gram-negative bacteria, among which compound **10** demonstrated strong and very strong bioactivity toward the tested Gram-negative bacteria. Furthermore, compound **10** was found to possess very strong efficacy against a wide range of β-lactamases producing strains, especially when combined with standard antibiotic ceftazidime. Our research revealed that further optimization of imidazole 2-aldoxime derivatives can result with greater specificity and efficacy for the treatment of multidrug resistant bacterial infections.

## Figures and Tables

**Table 1:** Antimicrobial activity of *N*-substituted imidazole 2-aldoximes (**1**–**5**) and their quaternary salts (**6**–**11**) by disc diffusion assay.

Microorganisms	Diameters of the Inhibition Zone (mm)											
	Compound No ^a^											Antibiotic ^b^
	1	2	3	4	5	6	7	8	9	10	11	GEN
**Gram-positive bacteria**												
*Bacillus cereus*	16.4 ± 0.3	15.4 ± 0.5	11.0 ± 0.5	13.3 ± 0.8	13.6 ± 0.8	19.2 ± 0.5	16.2 ± 0.2	19.1 ± 0.6	12.1 ± 0.6	21.2 ± 0.6	13.6 ± 0.1	18.2 ± 0.7
*Enterococcus faecalis*	17.7 ± 1.2	19.2 ± 0.4	16.2 ± 0.6	10.7 ± 0.6	12.7 ± 0.5	14.7 ± 0.7	20.4 ± 0.6	12.3 ± 0.7	20.3 ± 0.4	16.3 ± 0.2	14.5 ± 0.7	14.6 ± 1.4
*Staphylococcus aureus*	16.1 ± 0.9	15.1 ± 0.2	14.4 ± 0.2	12.3 ± 0.5	16.3 ± 2.4	16.1 ± 0.5	15.6 ± 1.6	19.7 ± 0.3	20.8 ± 0.7	16.7 ± 0.9	12.1 ± 0.8	23.9 ± 0.9
*Clostridium perfringens*	15.5 ± 0.2	11.7 ± 0.6	17.6 ±1.4	11.2 ± 0.8	14.4 ± 1.1	13.9 ± 1.2	17.7 ± 0.9	19.4 ± 0.4	15.2 ± 0.3	14.1 ± 1.0	15.9 ± 0.2	21.7 ± 0.4
**Gram-negative bacteria**												
*Escherichia coli*	18.8 ± 0.3	18.9 ± 0.2	21.8 ± 0.9	17.3 ± 1.1	18.3 ± 0.4	20.7 ± 0.5	17.1 ± 0.9	20.7 ± 0.8	12.4 ± 0.7	20.4 ± 1.1	13.4 ± 0.7	11.5 ± 0.9
*Klebsiella pneumoniae*	15.2 ± 1.5	16.3 ± 0.1	17.3 ± 0.0	14.8 ± 0.3	16.5 ± 0.1	20.1 ± 0.8	17.5 ± 0.8	19.4 ± 0.2	14.1 ± 0.3	18.2 ± 0.8	16.8 ± 0.5	18.8 ± 0.6
*Pseudomonas aeruginosa*	16.8 ± 1.9	18.9 ± 0.4	16.8 ± 0.3	14.9 ± 0.1	14.9 ± 0.3	20.2 ± 0.4	18.6 ± 0.4	16.7 ± 04	13.3 ± 0.7	18.8 ± 1.6	12.2 ± 1.1	9.7 ± 1.4
*Cronobacter sakazakii*	13.7 ± 1.5	15.7 ± 1.9	18.3 ± 0.1	13.1 ± 0.3	12.3 ± 0.8	13.6 ± 0.2	13.7 ± 0.1	16.1 ± 0.6	12.4 ± 0.1	16.4 ± 0.7	12.1 ± 0.6	13.6 ± 0.9

^a^ Diameter of inhibition zone (values in mm) around the disc: 200 μg/disc. ^b^ Standard antibiotic disc: **GEN**, gentamicin (15 μg/disc). All values are expressed as mean ± SD of three parallel measurements.

**Table 2:** The minimum inhibitory concentration of *N*-substituted imidazole 2-aldoximes (**1**–**5**), their quaternary salts (**6**–**11**) and gentamicin (**GEN**). Values in bold indicate very strong bioactivityof compounds.

MIC																								
	Compound No																						Antibiotic	
Microorganisms	1		2		3		4		5		6		7		8		9		10		11		GEN	
	µg/mL	μmol/L	µg/mL	μmol/L	µg/mL	μmol/L	µg/mL	μmol/L	µg/mL	μmol/L	µg/mL	μmol/L	µg/mL	μmol/L	µg/mL	μmol/L	µg/mL	μmol/L	µg/mL	μmol/L	µg/mL	μmol/L	µg/mL	μmol/L
**Gram-positive bacteria**																								
*Bacillus cereus*	25.00	163.2	50.00	299.0	50.00	302.7	25.00	106.1	25.00	106.1	**12.50**	**34.9**	25.00	69.7	25.00	67.1	25.00	67.1	25.00	67.5	**12.50**	**33.7**	4.00	8.4
*Enterococcus faecalis*	**12.50**	**81.6**	50.00	299.0	50.00	302.7	25.00	106.1	25.00	106.1	25.00	69.7	25.00	69.7	25.00	67.1	50.00	134.2	25.00	67.5	25.00	67.5	4.00	8.4
*Staphylococcus aureus*	50.00	326.4	25.00	149.5	25.00	151.3	**12.50**	**53.0**	25.00	106.1	25.00	69.7	**12.50**	**34.9**	25.00	67.1	50.00	134.2	25.00	67.5	50.00	134.9	1.00	2.1
*Clostridium perfringens*	25.00	163.2	12.50	74.8	25.00	151.3	25.00	106.1	12.50	53.0	50.00	139.4	**12.50**	**34.9**	25.00	67.1	50.00	134.2	25.00	67.5	**12.50**	**33.7**	0.50	1.1
**Gram-negative bacteria**																								
*Escherichia coli*	**12.50**	**81.6**	25.00	149.5	50.00	302.7	**12.50**	**53.0**	50.00	212.2	25.00	69.7	25.00	69.7	50.00	134.2	50.00	134.2	**6.25**	**16.9**	25.00	67.5	32.00	67.0
*Klebsiella pneumoniae*	25.00	163.2	25.00	149.5	50.00	302.7	25.00	106.1	25.00	106.1	25.00	69.7	25.00	69.7	25.00	67.1	50.00	134.2	**6.25**	**16.9**	25.00	67.5	8.00	16.8
*Pseudomonas aeruginosa*	25.00	163.2	50.00	299.0	25.00	151.3	25.00	106.1	50.00	212.2	25.00	69.7	25.00	69.7	50.00	134.2	50.00	134.2	**12.50**	**33.7**	**12.50**	**33.7**	64.00	134.0
*Cronobacter sakazakii*	25.00	163.2	25.00	149.5	25.00	151.3	25.00	106.1	50.00	212.2	50.00	139.4	25.00	69.7	50.00	134.2	50.00	134.2	25.00	67.5	25.00	67.5	8.00	16.8

**Table 3:** Minimum inhibitory concentration of quaternary imidazolium oximes **10** and **11** (µg/mL) against multidrug resistant Gram-negative ESBL-producing isolates. Values in bold indicate very strong bioactivity (MIC < 10 µg/mL) of compounds **10** and **11**.

Species, ESBL	MIC																			
	Compounds				Standard Antibiotics ^a^															
	10		11		CAZ		CTX		CIP		GEN		TET		TOB		IMP		MEM	
	µg/mL	μmol/L	µg/mL	μmol/L	µg/mL	μmol/L	µg/mL	μmol/L	µg/mL	μmol/L	µg/mL	μmol/L	µg/mL	μmol/L	µg/mL	μmol/L	µg/mL	μmol/L	µg/mL	μmol/L
*Klebsiella pneumoniae*, TEM-1, SHV-11, CTX-15	**4**	**10.8**	12	32.4	128	234.2	64	140.5	2.00	6.0	32	67.0	256	576.0	2.0	4.3	0.12	0.4	0.12	0.3
*Klebsiella pneumoniae*, TEM-1, CTX-15, AmpC	12	32.4	32	86.3	>128	>234.2	>256	>562.1	0.25	0.8	4	8.4	16	36.0	32	68.4	0.25	0.8	0.50	1.3
*Escherichia coli*, CTX-15	**8**	**21.6**	16	43.2	8	14.6	>256	>562.1	0.50	1.5	8	16.8	128	288.0	64	136.9	0.12	0.4	1.00	2.6
*Escherichia coli*, SHV-12, CTX-15	**4**	**10.8**	12	32.4	64	117.1	>256	>562.1	0.50	1.5	16	33.5	32	72.0	16	34.2	0.06	0.2	0.12	0.3
*Escherichia coli*, TEM-1, SHV-12	**2**	**5.4**	12	32.4	16	29.3	32	70.3	1.00	3.0	128	268.0	64	144.0	>32	>68.4	8	26.7	0.25	0.7
*Enterobacter cloacae*, SHV-12	12	32.4	**4**	**10.8**	8	14.6	8	17.6	0.50	1.5	2	4.2	16	36.0	>32	>68.4	0.12	0.4	0.12	0.3
*Enterobacter cloacae*, TEM-1, SHV-12, CTX-15	**2**	**5.4**	**8**	**21.6**	64	117.1	256	562.1	2.00	6.0	4	8.4	8	18.0	16	34.2	0.06	0.2	0.06	0.2
*Raultella terrigena*, TEM-1, SHV-11, CTX-15	**4**	**10.8**	32	86.3	4	7.3	256	562.1	0.50	1.5	128	268.0	64	144.0	32	68.4	0.25	0.8	0.125	0.3
*Citrobacter freundii*, TEM-1, SHV-12, CTX-15, AmpC	**8**	**21.6**	12	32.4	128	234.2	256	562.1	1.00	3.0	32	67.0	128	288.0	32	68.4	2.00	6.7	1.00	2.6
*Pantoea agglomerans*, CTX-15	16	43.2	**4**	**10.8**	16	29.3	256	562.1	0.25	0.8	64	134.0	16	36.0	32	68.4	0.25	0.8	0.12	0.3

^a^ Abbreviations of standard antibiotics: ceftazidime (**CAZ**), cefotaxime (**CTX**), ciprofloxacin (**CIP**), gentamicin (**GEN**), tetracycline (**TET**), tobramycin (**TOB**), imipenem (**IMP**) and meropenem (**MEM**).

**Table 4:** Combination study of antibiotic ceftazidime (**CAZ**) with compound **10** (**CAZ**:**10** mass ratio = 3:2, molar concentration ratio 1:1) against multidrug resistant Gram-negative ESBL-producing isolates. Values in bold indicate very strong synergy and bioactivity (MIC ≤ 1 µg/mL) of **10** + **CAZ** mixture.

Species, ESBL	MIC CAZ Alone		MIC 10 Alone		MIC Combination CAZ, 10	
	(μg/mL)	(μmol/L)	(μg/mL)	(μmol/L)	(μg/mL)	(μmol/L)
*Klebsiella pneumoniae*, TEM-1, SHV-11, CTX-15	128	234	4	11	4.8, 3.2	8.8, 8.6
*Klebsiella pneumoniae*, TEM-1, CTX-15, AmpC	>128	234	12	32	9.6, 6.4	17.6, 17.3
*Escherichia coli*, CTX-15	8	15	8	22	**0.6, 0.4**	**1.1, 1.1**
*Escherichia coli*, SHV-12, CTX-15	64	117	4	11	**0.3, 0.2**	**0.5, 0.5**
*Escherichia coli*, TEM-1, SHV-12	16	29	2	5	**0.6, 0.4**	**1.1, 1.1**
*Enterobacter cloacae*, SHV-12	8	15	12	32	**0.3, 0.2**	**0.5, 0.5**
*Enterobacter cloacae*, TEM-1, SHV-12, CTX-15	64	117	2	5	1.2, 0.8	2.2, 2.2
*Raultella terrigena*, TEM-1, SHV-11, CTX-15	4	7	4	11	2.4, 1.6	4.4, 4.3
*Citrobacter freundii*, TEM-1, SHV-12, CTX-15, AmpC	128	234	8	22	4.8, 3.2	8.8, 8.6
*Pantoea agglomerans*, CTX-15	16	29	16	43	**0.6, 0.4**	**1.1, 1.1**
